# bmo-miR-275 down-regulates expression of *Bombyx mori* sericin gene 2 in vitro

**DOI:** 10.1371/journal.pone.0190464

**Published:** 2018-01-30

**Authors:** Ping Qian, Tao Jiang, Xin Wang, Fei Song, Chen Chen, Xingjia Shen

**Affiliations:** 1 Jiangsu Key Laboratory of Sericultural Biology and Biotechnology, School of Biotechnology, Jiangsu University of Science and Technology, Zhenjiang, Jiangsu, China; 2 Key Laboratory of Silkworm and Mulberry Genetic Improvement, Ministry of Agriculture, Sericultural Research Institute, Chinese Academy of Agricultural Sciences, Zhenjiang, Jiangsu, China; Institute of Plant Physiology and Ecology Shanghai Institutes for Biological Sciences, CHINA

## Abstract

We hypothesized that bmo-miR-275 has a potential regulatory function regarding the expression of sericin gene 2 (*BmSer-2*). First, we examined the expression of bmo-miR-275 and its target gene *BmSer-2* in seven different tissues from 5th instar day-3 silkworm larvae. The results showed that they were both specifically expressed in the middle silk gland, implying that spatio-temporal conditions are required for bmo-miR-275 to regulate the expression of *BmSer-2*. To test this hypothesis, we constructed a pri-bmo-miR-275 expressing plasmid pcDNA3.0 [*ie1-egfp*-pri-bmo-miR-275-SV40] and *BmSer-2*-3´UTR recombinant reporter plasmids pGL3.0 [*A3-luc-Ser-2*-3′ UTR-SV40]. Finally, *Bm*N cells were harvested and luciferase activity was detected. Results showed that luciferase activity was reduced significantly (P<0.05) in *Bm*N cells co-transfected with pcDNA3.0 [*ie1-egfp*-pri-bmo-miR-275-SV40] and pGL3.0 [*A3-luc-Ser-2*-3’UTR-SV40], suggesting that bmo-miR-275 can down-regulate the expression of *BmSer-2* in vitro. Our results improve the understanding of the regulatory function of *Bombyx mori* miRNA on the expression of genes regulating silk formation.

## Introduction

MicroRNAs (miRNAs) are a class of endogenous noncoding regulatory RNAs with a length of 19–22 nucleotides. Mature miRNAs are sheared from longer primary transcripts through a series of nuclease cleavage and modification processes and are then assembled into RNA-induced silencing complexes. Mature miRNAs recognize target mRNAs through complimentary base-pairing and regulate silencing complexes according to the degree of complementarity with the target mRNA to degrade target mRNA or repress translation[1–4]. miRNAs are mostly found in clusters in the genome[5], and most sequences are located in intergenic regions, indicating that the transcription of miRNA is independent of other gene transcription and that these species have their own mechanisms for transcriptional regulation. Studies have shown that animal and plant miRNAs are located in the 3′ and 5′ untranslated regions (UTRs) of the target mRNA[6–8]. miRNAs exhibit time-dependent, tissue-specific expression, which is highly conserved[9]. Additionally, miRNAs are thought to regulate more than 30% of all genes, blocking the expression of genes involved in many physiological and pathological processes[10, 11].

*Bombyx mori* is an important model used for studying animal development, differentiation, growth, immune regulation, genetics, and mutations[12]. The silk gland of *B*. *mori* can synthesize silk proteins with high efficiency and specificity and has been the focus of research for many years. The silk gland of *B*. *mori* includes three parts: the anterior silk gland, middle silk gland, and posterior silk gland. The silk protein sericin is synthesized mainly in the middle silk gland. *BmSer-2* in 11 chromosome is a specific gene coding sericin protein expressing in 120 cells of front of middle silk gland and is consist of 12 exon and 11 intron. Huang et al[13, 14] showed that bmo-miR-2b can suppress the expression of the fibroin protein gene *BmP25* as well as bmo-miR-965 and bmo-miR-1926 can suppress the expression of the fibroin light chain gene (*BmFib-L*). Song Fei et al[15] demonstrated that bmo-miR-2739 can up-regulatethe fibroin heavy chain gene (*BmFib-H*). Additionally, we recently used Solexa to sequence the sRNA of the middle silk gland of wild-type (P50) and fifth instar, 3-day larvae and obtained several differentially expressed known miRNAs. Thus, we speculated that these miRNAs might be associated with the synthesis of the silk protein sericin.

In this study, we performed bioinformatics analysis to identify potential targets of bmo-miR-275 and subsequently analyzed the role of bmo-miR-275 in the regulation of *BmSer-2*. Our data provide important insights into miRNA functions in B. mori and the molecular mechanisms regulating the expression of genes encoding silk proteins.

## Materials and methods

### Total RNA extraction from tissues of *B*. *mori* larva

An RNAiso Plus kit (TaKaRa, Shiga, Japan) was used to extract total RNA from various tissues and organs of wild-type silkworms (P50) at 3-day fifth instar larvae provided by the Sericultural Research Institute, Chinese Academy of Agricultural Sciences (CAAS; Zhenjiang, China). The following tissues and organs were collected: skin, fat body, Malpighian tubules, midgut, anterior silk gland, middle silk gland, posterior silk gland, and head. Electrophoresis was used to determine the quality of total RNA.

### Prediction of miRNAs in *B*. *mori*

Differentially expressed miRNAs obtained from Solexa-based high-throughput sequencing of the middle silk gland in wild-type silkworms (P50) at fifth instar 3-day larvae in preliminary experiments were used for comparisons with the 3′UTR nucleic acid sequence of *BmSer-2* in *B*. *mori*, as downloaded from the NCBI database. Sequences of *B*. *mori* mature miRNAs from miRBase (http://www.mirbase.org/) were analyzed by RNAhybrid software (http://bibiserv.techfak.uni-bielefeld.de/rnahybrid). The potential targets of miRNAs were identified as having perfect sequence complementarity between the seed region of the miRNA (7-nucleotide sequence from bases 2 to 8 at the 5’ end of the miRNAs) and the 3’UTR of target mRNAs, and less than −20.0 kcal/mol (1 kcal/mol = 4.182 kJ/mol) free energy in the secondary structure of the miRNA/mRNA duplex[16].

#### Identification of bmo-miR-275

The specific reverse transcription primers for bmo-miR-275 were designed in accordance with a previous studies[17]. Total RNA from the middle silk gland of the fifth instar 3-day larvae was used as the template, and cDNA synthesis was performed using a reverse transcription kit (TaKaRa) following the manufacturer’s protocol. The cDNA was then used as a template for PCR. The PCR conditions were as follows: denaturation at 94°C for 5 mins, 30 cycles of 94°C for 30 s, 64°C for 30 s, and 72°C for 30 s,with a final extension at 72°C for 10 mins. PCR products were detected by electrophoresis using 4% agarose gels. The sequences of interest were collected and ligated into the pMD18-T vector at 16°C overnight. The ligation products were transformed into *E*.*coli* Top10 competent cells. The plasmid DNA was then extracted and sequenced. Sequencing results were compared using ClustalX 1.83 software.

### Semiquantitative RT-PCR for bmo-miR-275 and its predicted target genes in B. mori

#### Primer design and synthesis

The upstream/downstream primers for *BmSer-2*, primers for semiquantitative RT-PCR, and upstream/downstream primers for bmo-miR-275, pri-miR-275 (the primary of bmo-miR275) and the internal control U6 a housekeeping gene in *B*.*mori* were designed using Oligo 6.0 software. Primers were synthesized by Shanghai Biological Engineering Technical Services Corporation (Sangon, China).

#### cDNA synthesis and semiquantitative RT-PCR

Total RNA, extracted from different organs of B. mori larvae, was reverse transcribed into a cDNA template for bmo-miR-275, pri-miR-275, BmSer-2, and U6 amplification using specific primers and a reverse transcription kit (TaKaRa), according to the manufacturer’s protocol. The cDNA concentration of each sample was diluted to 500ng/μL as the template for PCR. The PCR conditions were as follows: denaturation at 94°C for 5 mins, 28 cycles of 94°C for 30 s,64°C for 30 s and 72°C for 30 s, with a final extension at 72°C for 10 mins.

### Construction of recombinant vectors expressing bmo-miR-275 and its predicted target genes

The pGL3.0-Basic plasmid was used as the template vector. The BmSer-2 3′UTR region was cloned downstream of the luciferase-encoding gene, luc, and the A3 promoter to generate the recombinant vector pGL3.0 expressing a fusion gene containing the BmSer-2 3′UTR and the luc gene (A3-luc-Ser-2-3′UTR-SV40). The pcDNA3 plasmid was used as the template vector. The fragment containing the enhanced green fluorescent protein-encoding gene, egfp, and the nucleotide sequences of precursors of the bmo-miRNA-275 and their up and down-stream flanking regions which were downloaded from SilkBase (http://silkbase.ab.a.u-tokyo.ac.jp/cgi-bin/index.cgi) were cloned into the vector with *Bm*NPV ie1 as the promoter to construct the recombinant vector expressing bmo-miR-275 pcDNA3 (ie1-egfp-pri-miR-275-SV40).

#### Plasmid transfection for verification of the regulation of predicted target gene expression by bmo-miR-275

Healthy growing BmN cells were seeded in 12-well cell culture plates 1 day before transfection. Then, 1 mL of cell suspension, gently pipetted from the flasks, was added per well for cell culture to allow cell attachment. The cell density for transfection was approximately 1 × 105 cells/mL, and the plasmid concentration was approximately 400 ng/μL. BmN cells were co-transfected as follows. The Renilla luciferase reporter plasmid pRL-CMV was used as an internal control. Two solutions were prepared in 1.5-mL centrifuge tubes; solution A contained 3 μL reporter plasmid DNA in 50 μL medium without antibiotics or fetal calf serum and solution B contained 1 μL transfection reagent Per-Fect (UCallM, China) dissolved in 50 μL medium without antibiotics or fetal calf serum. Solutions A and B were evenly mixed and incubated at room temperature for 30 min. The original cell culture solution in 12-well cell culture plates was discarded, and the cells were washed with 1 mL medium without serum or antibiotics. Washing medium was then discarded and 800 μL cell culture medium without antibiotics or fetal calf serum was added to the mixture of solutions A and B. The solutions were mixed well and then added to cell culture plates. Cells were incubated in a 37°C incubator for 5 h, after which the mixture of solutions A and B was removed, and 1 mL medium containing fetal calf serum and antibiotics was added. Cells were cultured at 37°C for 72 h and were then collected for luciferase assays. Each treatment was repeated three times. A LuminoMeter 20/20n fluorescence spectrophotometer (Turner Biosystems, USA) was used to measure activity of luciferase and Renilla luciferase[18, 19]. After correction using the negative control and blank control, the ratio of the two measurements was calculated to analyze the regulation of Ser-2 expression by bmo-miR-275. Data were analyzed for statistical significance using SPSS 16.0 software.

## Results

### Evaluation of the quality of total RNA from B. mori larvae tissues and the bmo-miR-275 precursor

The bmo-miR-275 mature sequence of B. mori was obtained through sequencing conducted by Liu et al[20]. Total RNA from tissues of the fifth instar 3-day larvae was extracted and determined to be of sufficiently good quality for subsequent use by electrophoresis using 2% agarose gels. All RNA samples showed three clear bands (data not shown).

**Fig 1:**
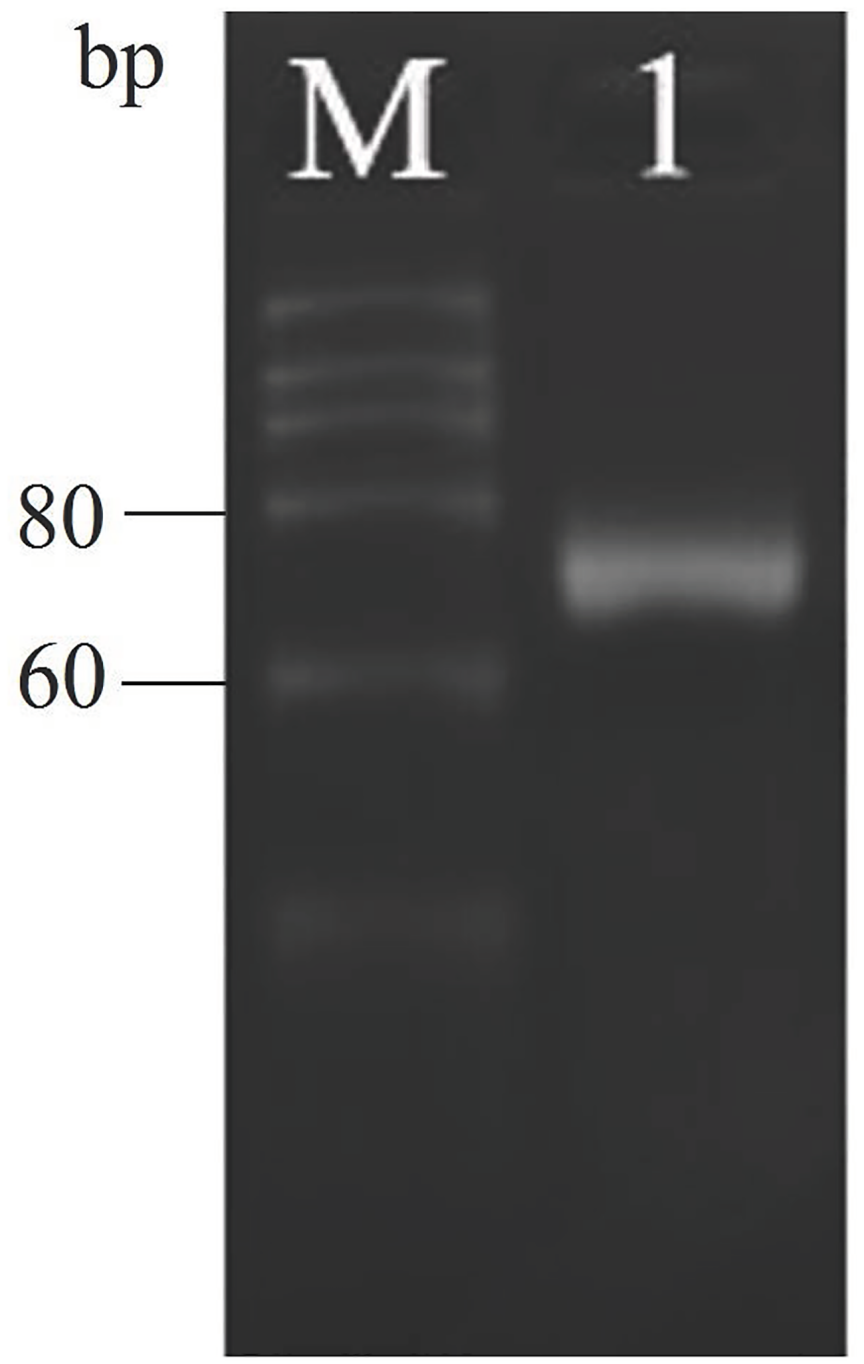
Electrophoresis pattern of RT-PCR products ofbmo-miR-275. (M: 20 bp marker;1: bmo-miR-275).

The cDNA templates for amplification were generated by reverse transcription from the total RNA of the middle silk gland of the fifth instar 3-day larvae. Electrophoresis showed a fragment with a length of 60–80 bp, which was consistent with the theoretical length of the bmo-miR-275 precursor fragment (Fig 1). The fragment of interest was collected and cloned into the pMD18-T vector, and the plasmid was then transformed into *E*. *coli* Top10 competent cells. The recombinant plasmid was extracted and sequenced. The size of the sequenced product coincided with that of the expected sequence.

### Prediction of bmo-miR-275 targeting BmSer-2 3’UTR

**Fig 2:**
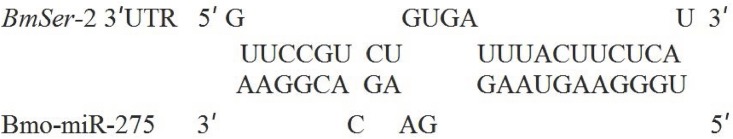
Prediction of the binding sites of bmo-miR-275 in *BmSer-2* mRNA using RNAhybrid software. The folding energy was −16.3 kcal/mol. The initiation binding site at the target gene was the 39th base of the 3’UTR.

Target prediction software RNAhybrid was used to assess the scores and the complementarity between the miRNA seed regions and the target site. bmo-miR-275 was predicted to have the potential to target BmSer-2 3’UTR. Moreover, bmo-miR-275 binds *BmSer-2* 3’UTR starting from the 39th base and the folding energy is −16.3 kcal/mol, suggesting that bmo-miR-275 function independently (Fig 2).

### Temporal and spatial characteristics of the expression of bmo-miR-275 and its target gene in *BmSer-2*

**Fig 3:**
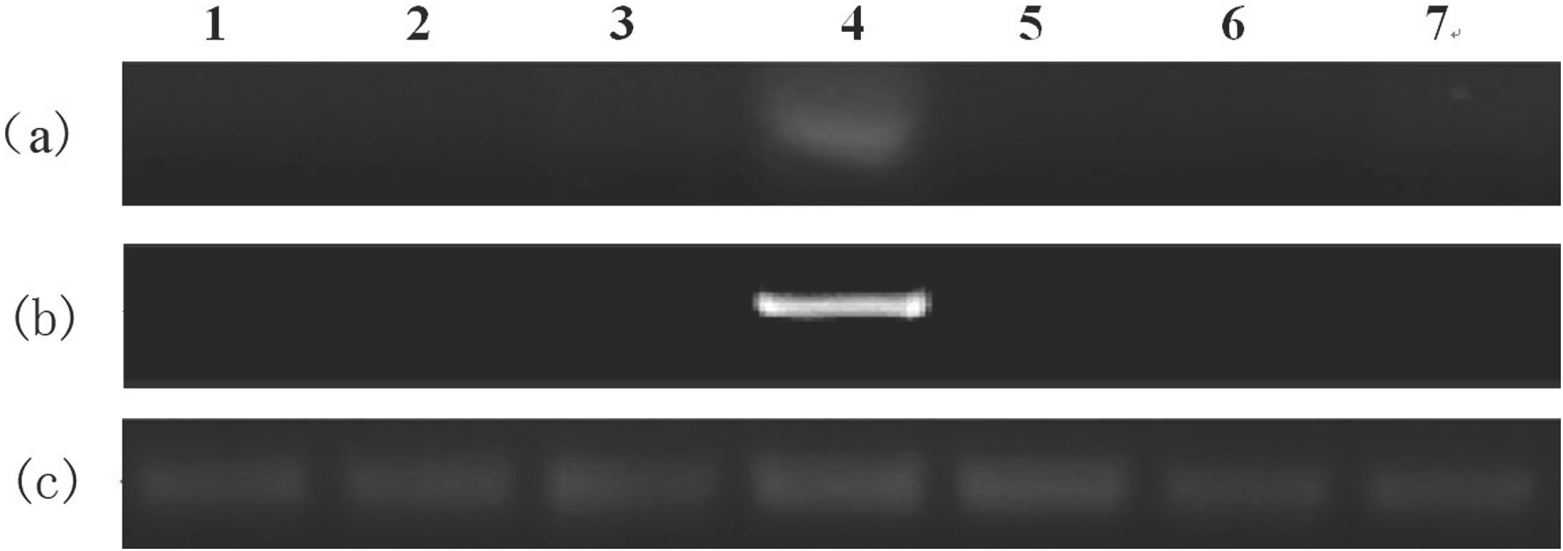
Expression analyses of bmo-miR-275, *BmSer-2*, and U6 in different tissues of 5th instar,3-day larvae of *B*. *mori*. (A) Expression of bmo-miR-275; (B) Expression of *BmSer-2*; (C) Expression of internal gene U6. 1, head; 2, fat body; 3,anterior silk gland;4,middle silk gland; 5, post silk gland; 6, testis;7, ovary.

The expression levels of bmo-miR-275 and its target gene *BmSer-2* in different tissues and organs of the fifth instar, 3-day larvae were detected using semi-quantitative reverse transcription polymerase chain reaction (RT-PCR)(The primers are list in Table 1). BmSer-2 was expressed only in the middle silk gland, but was not expressed in other tissues. In contrast, bmo-miR-275 was expressed only in the middle silk gland (Fig 3). These results suggest that the expression of bmo-miR-275 and *BmSer-2* in the fifth instar 3-day larvae was highly tissue specific.

**Table 1:** Primers used in this study.

Gene		Primer sequences (5′-3′)
**bmo-miR-275**	**RT**	GTCGTATCCAGTGCAGGGTCCGAGGTATTCGCACTGGATACGACTAACTA
	**Forward**	CGGGCTAACATTACGAGGA
	**Reverse**	GTGCAGGGTCCGAGGT
***BmU6***	**RT**	GTCGTATCCAGTGCAGGGTCCGAGGTATTCGCACTGGATACGACACG
	**Forward**	CCTGCGCAAGGATGAC
	**Reverse**	GTGCAGGGTCCGAGGT
**pri-mir-275**	**Forward**	CCCAAGCTTATTGGCCGAAATAAAATGAC
	**Reverse**	CGCGGATCCACACGACTGTTTGCGAAGGT
***BmSer-2***	**Forward**	CTTGTGGGCGTGGCTGTGG
	**Reverse**	AGCCGTCGCTGTCGTTATCCT

### Regulation of BmSer-2 expression by bmo-miR-275

#### Construction of expression vectors

**Fig 4:**
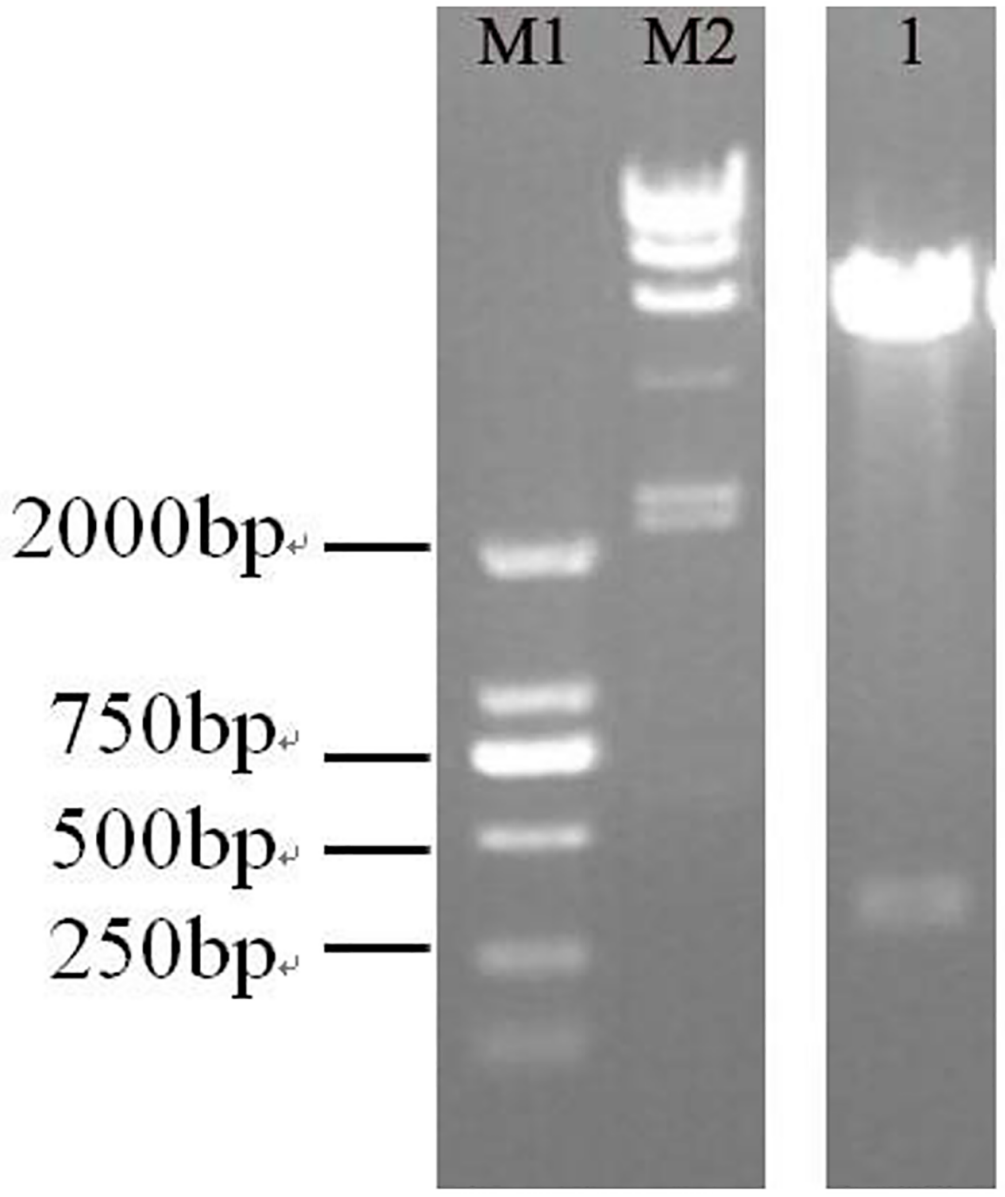
Double enzyme digestion of recombinant plasmid pcDNA3.0 [*ie*1-*egfp*-pri-bmo-miR-275-SV40] with *Hin*dIII and *Bam*HI. M1: DL 2000 DNA marker; M2:λ-HindШ Marker; 1: pcDNA3.0 [*ie*1-*egfp*-pri-bmo-miR-275-SV40] after double digestion with *Hin*dIII and *Bam*HI.

The expression vector pcDNA3.0 [*ie1-egfp*-pri-bmo-miR-275-SV40] for miR-275 expression was successfully constructed to contain the *Bm*NPV ie1 promoter and an egfp reporter gene. The inserted gene fragments were tested by electrophoresis using agarose gels after double digestion with *Hind*III and *BamH*I (Fig 4) and sequencing by Shanghai Biological Engineering Technical Services Corporation (Sangon,China).

### Co-transfection efficiency of recombinant expression vectors

In the treatment group, *Bm*N cells were co-transfected with a mixture of pcDNA3.0 [*ie*1-*egfp-*pri-bmo-miR-275-SV40], pGL3.0 [*A*3-*luc*-*Ser-2*-3’UTR-SV40], and pRL-CMV. In the control group, *Bm*N cells were co-transfected with a mixture of pcDNA3.0 [*ie*1*-egfp*-SV40], pGL3.0 [*A*3-*luc-Ser-2-*3’UTR-SV40], and pRL-CMV.

**Fig 5:**
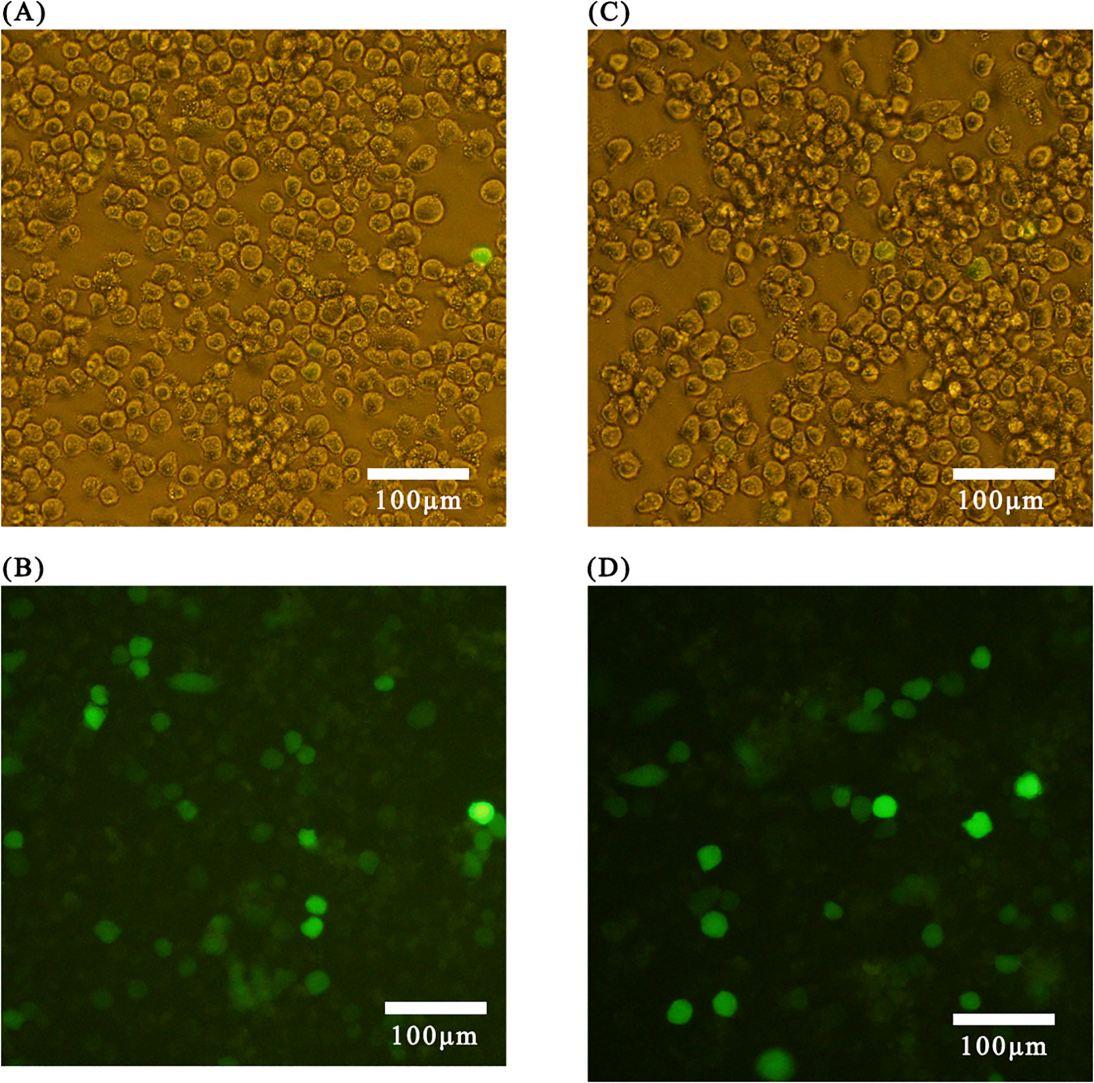
Expression of enhanced green fluorescent proteinin BmN cells transfected with recombinant plasmids. (A) pcDNA3.0 [*ie*1-*egfp*-SV40]+pGL3.0 [*A*3-*luc*-*Ser-2*-3’UTR-SV40]+pRL-CMV, bright light; (B) pcDNA3.0 (*ie*1*-egfp*-SV40)+ pGL3.0 (*A3-luc-Ser-2-*3’UTR-SV40)+pRL-CMV, fluorescence; (C) pcDNA3.0 (*ie*1-*egfp*-pri-bmo-miR-275-SV40)+ pGL3(*A3-luc*-*Ser-2-*3’UTR-SV40)+ pRL-CMV, brightlight; (D) pcDNA3.0 (*ie*1-*egfp*-pri-bmo-miR-275-SV40)+ pGL3(*A3-luc*-*Ser-2-*3’UTR-SV40)+ pRL-CMV, fluorescence.

Observations were performed using an inverted fluorescence microscope. Results showed that recombinant plasmids had been transfected into cells and that the majority of cells emitted green fluorescence (Fig 5).

#### Regulation of BmSer-2 by bmo-miR-275, as determined by luciferase activity assays

**Fig 6:**
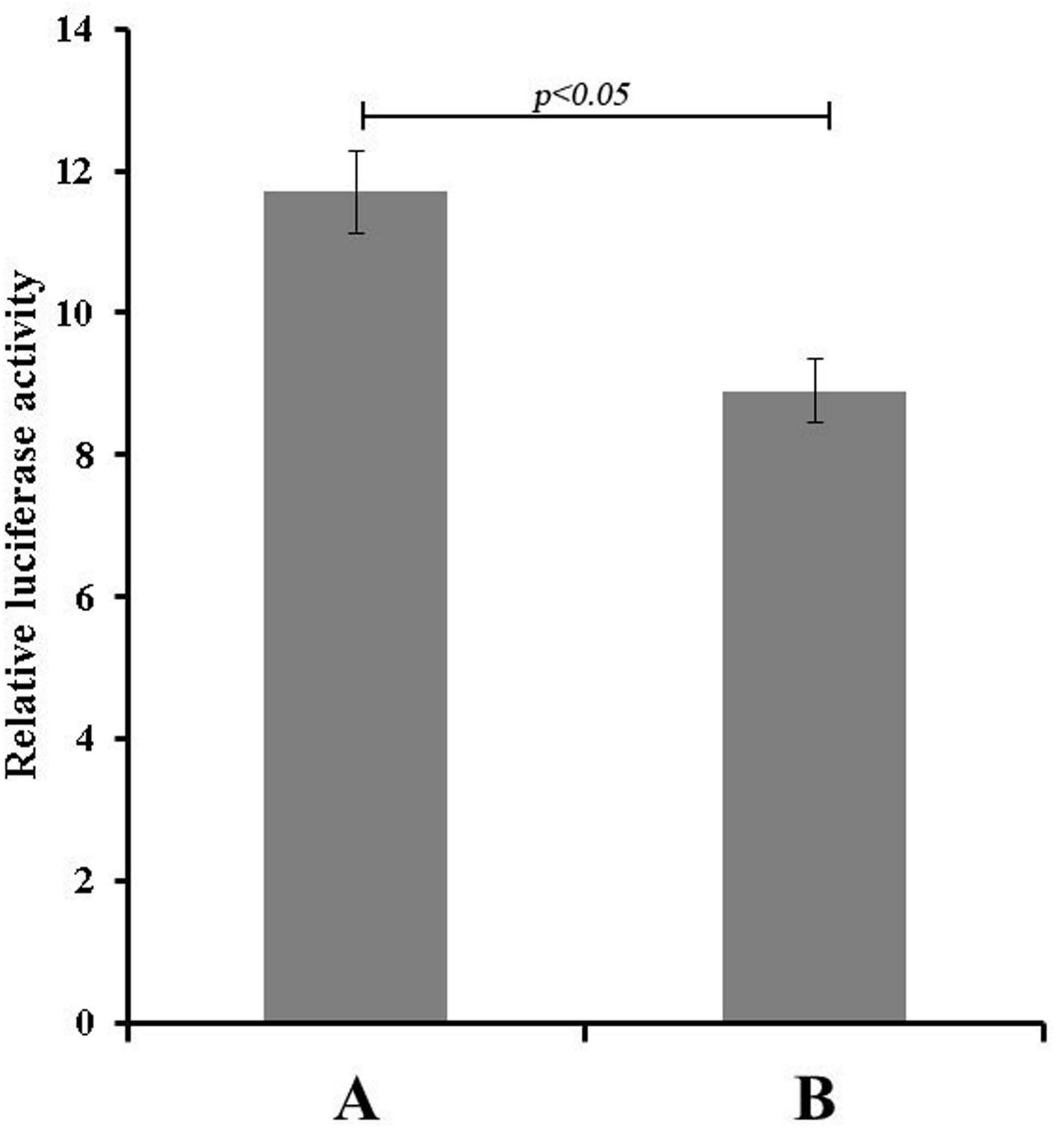
Effect of bmo-miR-275 expressionon luciferase activity in transfected *Bm*N cells. Data are represented as the mean ±standard deviation (SD) from three independent experiments. (A) pcDNA3.0 (*ie-1-egfp*-SV40)+ pGL3 (*A3-luc*-*Ser-2-*3’UTR-SV40)+pRL-CMV (B) pcDNA3.0 (ie-1-*egfp* -pri-mir-275-SV40)+ pGL3(*A3-luc*-*Ser-2-*3’UTR-SV40)+ pRL-CMV.

*Bm*N cells were co-transfected with the bmo-miR-275 recombinant expression vector and the recombinant expression vector containing the 3′UTR sequence of the target gene. Subsequent luciferase assays showed that luciferase activity in cells co-transfected with the bmo-miR-275 recombinant expression vector and the expression vector containing the 3′UTR of *BmSer-2* fused with the reporter gene was significantly decreased by nearly 29% (p < 0.05) compared to that in the control group (Fig 6).

From the above results, we conclude that*BmSer-2*is one of the target genes of bmo-miR-275, and that bmo-miR-275 can down-regulate the expression of the *BmSer-2* gene by interacting with the 3′UTR of its mRNA in vitro.

## Discussion

miRNAs participate in a variety of biological processes including cell proliferation, embryonic development, individual growth, disease initiation, and cell apoptosis[21–23]. Currently, there are 563 mature miRNA sequences in B. mori recorded in the miRBase database, indicating that miRNAs play important roles in the regulation of gene expression during the growth and development of silkworms. In this study, we used bioinformatics analysis and found that BmSer-2 mRNA is a target of bmo-miR-275. We showed, for the first time, that this miRNA significantly inhibited the expression of BmSer-2 using a dual luciferase reporter gene detection system. Our results not only enrich the understanding of the *B*. *mori* miRNA database, but also provide new experimental data for further studies on miRNA function and to elucidate the regulatory mechanisms of silk protein synthesis.

Some reports have shown that miRNAs can be regulated through the functions of competitive endogenous RNAs (ceRNAs)[24, 25]. These ceRNAs can act like “sponges” to absorb miRNA and reduce the concentration of miRNAs that are capable of binding to target mRNA in cells. This dampens miRNA inhibition and thereby increases the level of mRNA translation[26]. Based on this concept, rather than silk gland cells, we used BmN cells in experiments; these cells originated from the B. mori ovaries, which lack appropriate endogenous miRNA, permitting the binding of exogenous miRNAs to target mRNAs, thereby downregulating the expression of target genes. However, in vitro experimental models do not perfectly replicate the in vivo situation, so additional studies are needed to further validate the functions of miRNAs. This could be achieved by constructing recombinant baculoviral expression vectors for overexpression of B. mori miRNA and artificially synthesizing miRNA analogs or antisense RNA of miRNAs for transfection or injection into fifth instar larvae.

The regulatory mechanisms of silk protein expression are complicated, and the relationships between miRNAs and target genes can vary, with miRNAs regulating more than one target gene and a single target gene being regulated by more than one miRNA. Therefore, analysis of transcriptional regulation through the assessment of individual miRNAs is not sufficient for understanding the sophisticated mechanisms of silk protein gene regulation. Additional studies involving an increased number of transcription factors are needed to obtain more information regarding the regulation of silk protein gene expression by miRNAs. Furthermore, due to miRNA can bind to both the 3′ and 5′UTRs of mRNAs, the latter of which can promote gene expression rather than inhibit gene expression[27], more studies are needed to obtain information regarding target sites for miRNAs in the 5′UTRs of potential mRNA targets.
